# Screening for chronic kidney disease of uncertain aetiology in Sri Lanka: usability of surrogate biomarkers over dipstick proteinuria

**DOI:** 10.1186/s12882-017-0610-x

**Published:** 2017-06-19

**Authors:** Samantha Ratnayake, Zeid Badurdeen, Nishantha Nanayakkara, Tilak Abeysekara, Neelakanthi Ratnatunga, Ranjith Kumarasiri

**Affiliations:** 1 Center for Research and Training on Kidney Diseases (CERTKiD), Faculty of Medicine, University of Peradeniya, Peradeniya, Sri Lanka; 2 Sri Lanka Institute of Nanotechnology (SLINTEC), Homagama, Sri Lanka; 3 Transplant and Dialysis unit, Teaching Hospital, Kandy, Sri Lanka; 4 Department of Pathology, Faculty of Medicine, University of Peradeniya, Peradeniya, Sri Lanka; 5 Department of Community Medicine, Faculty of Medicine, University of Peradeniya, Peradeniya, Sri Lanka

**Keywords:** CKDu, Proteinuria, Biomarkers

## Abstract

**Background:**

The use of dipstick proteinuria to screen Chronic Kidney Disease of uncertain aetiology (CKDu) in Sri Lanka is a recently debated matter of dispute. The aim of this study was to assess the suitability of biomarkers: serum creatinine, cystatin C and urine albumin to creatinine ratio (ACR) for screening CKDu in Sri Lanka.

**Methods:**

Forty-four male CKDu patients and 49 healthy males from a CKDu-endemic region were selected. Meanwhile, 25 healthy males from a non-endemic region were selected as an absolute control. The diagnostic accuracy of each marker was compared using the above three study groups.

**Results:**

In receiver operating characteristics (ROC) plots for creatinine, cystatin C and ACR, values of area under the curve (AUC) were 0.926, 0.920 and 0.737 respectively when CKDu was compared to non-endemic control. When CKDu was compared to endemic control, AUCs of above three analytes were distinctly lower as 0.718, 0.808 and 0.678 respectively. Cystatin C exhibited the highest sensitivity for CKDu when analyzed against both control groups where respective sensitivities were 0.75 against endemic control and 0.89 against non-endemic control. ROC-optimal cutoff limits of creatinine, cystatin C and ACR in CKDu vs non-endemic control were 89.0 μmol/L, 1.01 mg/L and 6.06 mg/g-Cr respectively, whereas in CKDu vs endemic control the respective values were 111.5 μmol/L, 1.22 mg/L and 12.66 mg/g-Cr.

**Conclusions:**

Amongst the three biomarkers evaluated in this study, our data suggest that Cystatin C is the most accurate functional marker in detecting CKDu in endemic regions, yet the high cost hinders its usability on general population. Creatinine is favorable over dipstick proteinuria owing to its apparent accuracy and cost efficiency, while having the ability to complement the kidney damage marker (ACR) in screening. ACR may not be favorable as a standalone screening marker in place of dipstick proteinuria due to its significant decline in sensitivity against the CKDu-endemic population. However, creatinine and ACR in a complementary manner could overcome current shortcomings of dipstick proteinuria and such a dual marker tool could be commodious in screening CKDu-type tubulointerstital diseases. Furthermore, use of ACR may also increase the ability to clinically discriminate CKDu from other glomerular nephropathies.

## Background

A remarkable increase in renal diseases has been observed during the time period between 1990 and 2007 in Sri Lanka. Around the year 2000, it was observed that the number of CKD patients from the North Central Province (NCP) has risen and a retrospective study at that time reported a new form of CKD which was not associated with conventional risk factors [1]. Due to the illusive nature of its aetiological factors it has been named “Chronic Kidney Disease of uncertain aetiology” (CKDu). CKDu is not limited to Sri Lanka; similar cases have been discovered in El Salvador, Nicaragua, Costa Rica, Srikakulam District in Andhra Pradesh, India and the Balkan region [2–10]. Athuraliya et al. (2011) reported that in Sri Lanka, CKDu is regionally biased towards the North central region of the country [11]. Anuradhapura, Polonnaruwa and parts of Badulla district have been identified as CKDu-endemic regions with higher incidence and prevalence of CKDu in Sri Lanka [11]. The initial CKDu screening tool, the semi-quantitative dipstick proteinuria, has detected macro albuminuria range in late-stage CKDu cases. However, accumulating evidence has demonstrated that this biomarker is not optimal to detect CKDu in early stages. Dipstick proteinuria based observational studies on prevalence of CKDu among a large number of populations show that it lacks in accuracy to be implemented into routine CKDu management [12].

CKDu is clinically defined as kidney damage in the absence of a past history of diabetes mellitus, chronic or severe hypertension, snake bite with systemic envenomation, glomerular nephritis or obstructive nephropathies. Presence of renal dysfunction when HbA1c < 6.5%, blood pressure < 160/100 mmHg untreated or <140/90 mmHg on up to two antihypertensive medications, residing in a CKDu endemic area for more than five years, exhibition of bilateral echogenic kidneys and a renal biopsy indicating a pathology of tubular interstitial disease can be denoted as demarcating parameters of CKDu [13]. Histopathological studies have reported that CKDu features tubular lesions as the major pathological characteristic while glomerular and vascular lesions are predominant in hypertensive or diabetic CKD [14]. The cause of CKDu in Sri Lanka is unknown; Nevertheless, subsequent studies suggest that the cause for CKDu in NCP might be an environmental factor, possibly related to drinking water or food [1, 12, 13, 15–17].

In the absence of known etiology, intervention in the early stages and modification of known CKD risk factors seems to be more effective to prevent and delay the progression to End Stage Renal Disease (ESRD). The protein detecting urine dipstick method in CKD screening is a widely used screening tool supported by many studies [11, 18, 19]. Proteinuria is an established marker in CKD diagnosis, progression and prediction of cardiovascular complications [20]. This is an inexpensive and rapid point-of-care diagnostic test that has high specificity and around 40% sensitivity in detecting proteinuria [21]. CKDu endemic populations were screened using dipstick proteinuria at the time of the study. Dipstick positive cases were confirmed with ACR, serum creatinine (S.Cr), renal ultrasound scan and renal biopsy in the detailed assessment.

The limitations of dipstick test include a high false-positive rate due to variation in individual reading, difficulty in getting early-morning first-void urine for testing as well as orthostatic proteinuria in untimed spot urine samples [22]. Moreover, nephrologists who are engaged in CKDu screening have observed early CKDu in Sri Lanka as a minimally-proteinuric disease exhibiting a sub-nephrotic range like other tubulointerstitial nephropathies. Due to these limitations, dipstick-negative subjects at preliminary screenings were later detected incidentally at an advanced stage challenging the initial screening process [23]. Nanayakkara et al. (2012) reported manifestation of elevated urinary tubular protein: α1-microglobulin in early CKDu in Sri Lanka. Similarly, low-molecular-weight proteins were detected in Chinese herbal nephropathy, Dent’s disease and some other forms of tubular diseases [24–26]. Majority of patients in the north central region of the country were screened with predominantly albumin detecting dipstick and were found to be positive in stages four and five at the detection [12]. Hence, it is evident that a more efficient, sensitive and a quantitative screening marker instead of urine dipstick proteinuria is required for early detection of the disease to achieve improved patient safety and reduced morbidity. No previous study has attempted to evaluate screening markers for CKDu in Sri Lanka. This study was designed to explicitly evaluate the usability of alternative CKD biomarkers in screening and diagnosis of CKDu in Sri Lanka.

In our study, efficiency of three biomarkers, serum cystatin C (S.Cys), and S.Cr as functional serum markers as well as urine ACR as a renal damage marker were tested on CKDu patients and two control groups. Among the biomarkers evaluated, ACR is a well-accepted, widely available, point of care marker, which is sensitive for low levels of albumin and it has been used as a successful biomarker for screening CKD in Indo-Asian populations by Jafar et al. in 2007 [27]. Further, it has been shown to be capable of detecting early stage CKD and effective in patients with hypertension or diabetes [28]. Even though urine based tests are more convenient as screening tests in field clinics, albumin or protein based tests can produce negative results even in advanced non-proteinuric types of tubular interstitial diseases. Due to this fact, we considered two serum based markers: Creatinine and Cystatin C. Creatinine is a breakdown product of a non-enzymatic process involving creatine phosphate and is a well-recognized endogenous marker in diagnosis and determination of the progression of CKD [29, 30]. Cystatin C is a small plasma protein molecule that is freely filtered at kidney glomerulus [31, 32]. It is a sensitive biomarker of kidney function in mild-to-moderate kidney disease. S.Cys was utilized as studies suggest it to be a superior indicator of estimated Glomerular Filtration Rate (eGFR) in comparison to S.Cr while being independent from age and sex-associated conditions [33–36]. A general overview of characteristics of the biomarkers in concern is presented in Table 1.

**Table 1:** A qualitative comparison of biomarkers of this study

Dipstick proteinuria	Creatinine	Cystatin C	ACR
Point-of-care test	Laboratory	Laboratory	Laboratory
Semi quantitative	Quantitative	Quantitative	Quantitative
Non-invasive	Minimally-invasive	Minimally-invasive	Non-invasive
Interpreter bias	None	None	None

## Methods

**Fig. 1:**
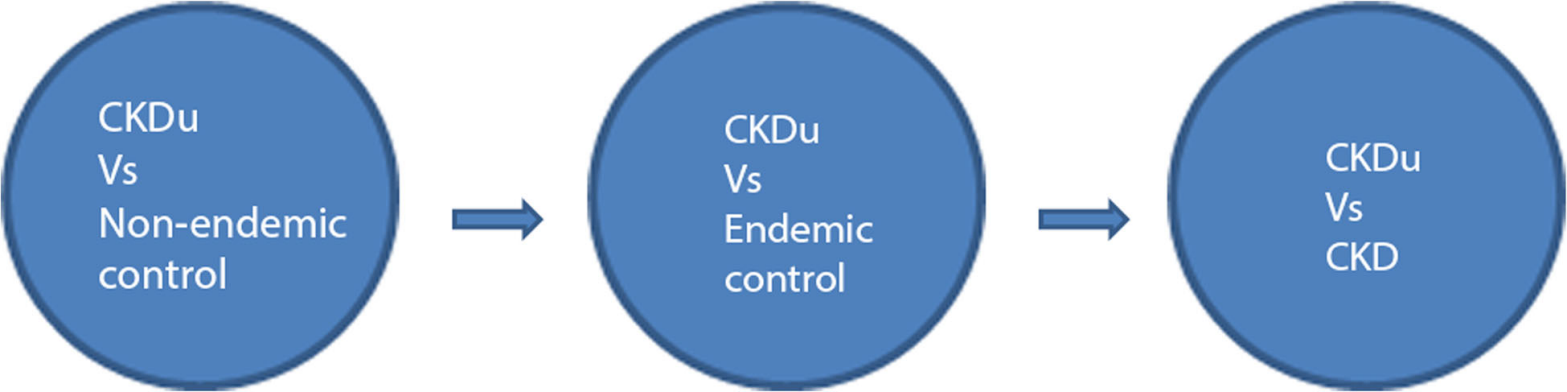
Sequence of study objectives

This study, as depicted in Fig. 1, sequentially attempts to discriminate CKDu from true controls followed by endemic controls and finally general CKD patients. Potential alternative biomarkers (S.Cr, S.Cys and ACR: hereafter denoted as target markers) were tested for all above mentioned patient and control groups alongside dipstick proteinuria.

Patient data was gathered based on a systematic screening strategy. Forty-four biopsy-proven male CKDu cases were selected as the patient group. Those patients had been initially referred to the renal clinic either from population screening programs or presented to clinicians at acute interstitial nephritis stage [23]. Hospital records were used to select a subset of patients such that a random demographic distribution was obtained. Voters’ lists were used to select 49 endemic controls to obtain a similar representation from the same regions. 25 non-endemic controls were similarly selected from a CKDu non-endemic area. In particular, both control groups had insignificant medical history and normal blood pressure values. All control subjects went through a routine screening protocol including detailed medical history to exclude subjects with renal diseases. Endemic controls were considered as ‘at risk controls’ whereas non-endemic controls were taken as ‘true controls’. Renal biopsy was taken as the gold standard for diagnosis of CKDu patients. Thirty CKD patients were selected for a comparative ROC analysis against CKDu where 9, 13 and 8 patients were selected from stage two, three and four respectively who represented proportions of different CKD etiologies in Sri Lanka according to a recent study [30]. CKD aetiologies were such that 11, 7 and 12 patients were from diabetic nephropathy, hypertensive nephropathy and other renal disorders respectively [all CKD patients were under Renin Angiotensin- Aldosterone System (RAAS) blockade]. General CKD patients were not subjected to further statistical analysis, therefore, the data is provided as a supplementary repository.

Blood samples for serum analysis were collected from a peripheral vein. Early morning first-void urine samples were collected in to polypropylene tubes and immediately stored at 4 °C after acquisition and transported to the laboratory. Laboratory analyses were performed at ISO 15189: 2007 certified Durdans laboratories, Sri Lanka LTD (Accreditation No: ML 010-01). S.Cys concentrations were measured by particle enhanced immuno-turbidimetry using the Dakocytomation assay kit (DAKO Ltd., Code No. LX002, Denmark, Standardized against ERM-DA471/IFCC). Creatinine was determined colorimetrically using picric acid based Jaffe’s reaction method (non IDMS-traceable). HbA1c was determined using a Bio-Rad D-10 HPLC analyzer while Urinary ACR was measured by Hitachi 911 and 912 auto-chemistry analyzers.

Statistics are reported as mean and standard deviation (SD), for frequency of continuous data. The Pearson’s correlation coefficient was used to evaluate the correlation between variables. Endemic and non-endemic control groups were subjected to an independent sample T-test. ROC curves were used to determine the clinical accuracy of target biomarkers. ROC plots were constructed and AUCs with 95% Confidence Interval (CI), sensitivity (Sn) and specificity (Sp) were calculated. An additional ROC plot was generated comparing CKDu patients and an equal number of stage- matched CKD patients. Optimal cutoff values for discrimination between the positive and negative diagnosis were set. Statistical analysis was performed using the SPSS software version 18.0 for Windows.

## Results

Characteristics of the three examined study groups are shown in Table 2. Systolic and diastolic blood pressures were normal and comparable among all groups. Meanwhile, the age compositions were similar among CKDu cases and non-endemic controls. Stage of renal disease computed by the Modified Diet for Renal Diseases (MDRD) formula indicated that 75% of the patients belonged to stage two and three. HbA1c levels were comparable only among the two control groups. Mean values for target biomarkers measured for each study group is given in Table 3 with reference ranges used in Sri Lanka.

**Table 2:** Case/control stratified characteristics of the study groups

	CKDu (SD)	Non-endemic control (SD)	Endemic control (SD)
Number (N)	44	25	49
Mean age (Years)	47.6 (8)	47.8 (13)	41.4 (9)
Mean eGFR (ml/min/1.73m^2^)	60.5 (23.4)	107 (25)	79 (16)
Mean BP^a^-Systolic (mmHg)	114.6 (16)	118.1 (16.5)	115.5 (14.3)
Mean BP-Diastolic (mmHg)	76 (10)	76.6 (8.4)	75.3 (8.5)
Mean HbA1c (%)	5.71 (0.38)	5.18 (0.27)	5.15 (0.41)

^a^Blood pressure

**Table 3:** Mean values of target biomarkers stratified by the study group

Parameter with reference value	CKDu^*a*^ (SD)	Non-endemic control^b^ (SD)	Endemic control^c^ (SD)
S.Cr (53–116 μmol/L)	140.1 (66.8)	74.1 (13.7)	102.5 (35.4)
S.Cys (0.59–1.03 mg/L)	1.7 (0.7)	0.86 (0.2)	1.08 (0.4)
ACR (<30 mg/g-Cr)	88.6 (177)	8.6 (13.4)	8.2 (8)

^a^CKDu patients from CKDu endemic regions (Giradurukotte)

^b^Controls from CKDu non-endemic regions

^c^Controls from CKDu endemic regions (Giradurukotte)

**Fig. 2:**
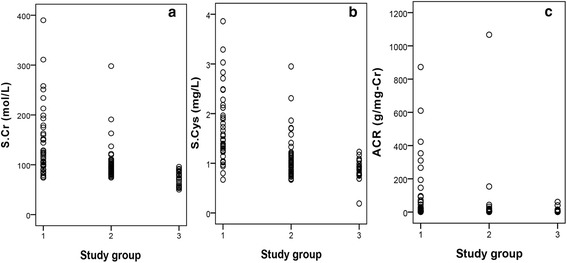
Individual values of: **a** S.Cr, **b** S.Cys and **c** ACR in patients and controls. Study group 1 – CKDu, 2 – Endemic control, 3 – Non-endemic control

S.Cr values of 88% of CKDu patients were above the current reference level for CKD. For S.Cys and ACR it was 95% and 32% respectively. S.Cr and S.Cys seems to work in an analogous manner. Non-endemic controls exhibited homogeneity and total diagnostic negativity in terms of S.Cr. However, two and five individuals in non-endemic control group were indicated as positive by ACR and S.Cys respectively. The mean S.Cr and S.Cys were observed lowest among non-endemic controls. Figure 2 depicts dispersion plots drawn for all measured parameters for the three study groups.

Among CKDu patients, a noticeably wider distribution of target markers was observed and is represented as a higher SD for CKDu group in Table 3. In terms of all target markers, non-endemic controls expressed a highly homogeneous pattern whilst, endemic controls exhibited a medium distribution falling in between CKDu cases and non-endemic controls.

**Fig. 3:**
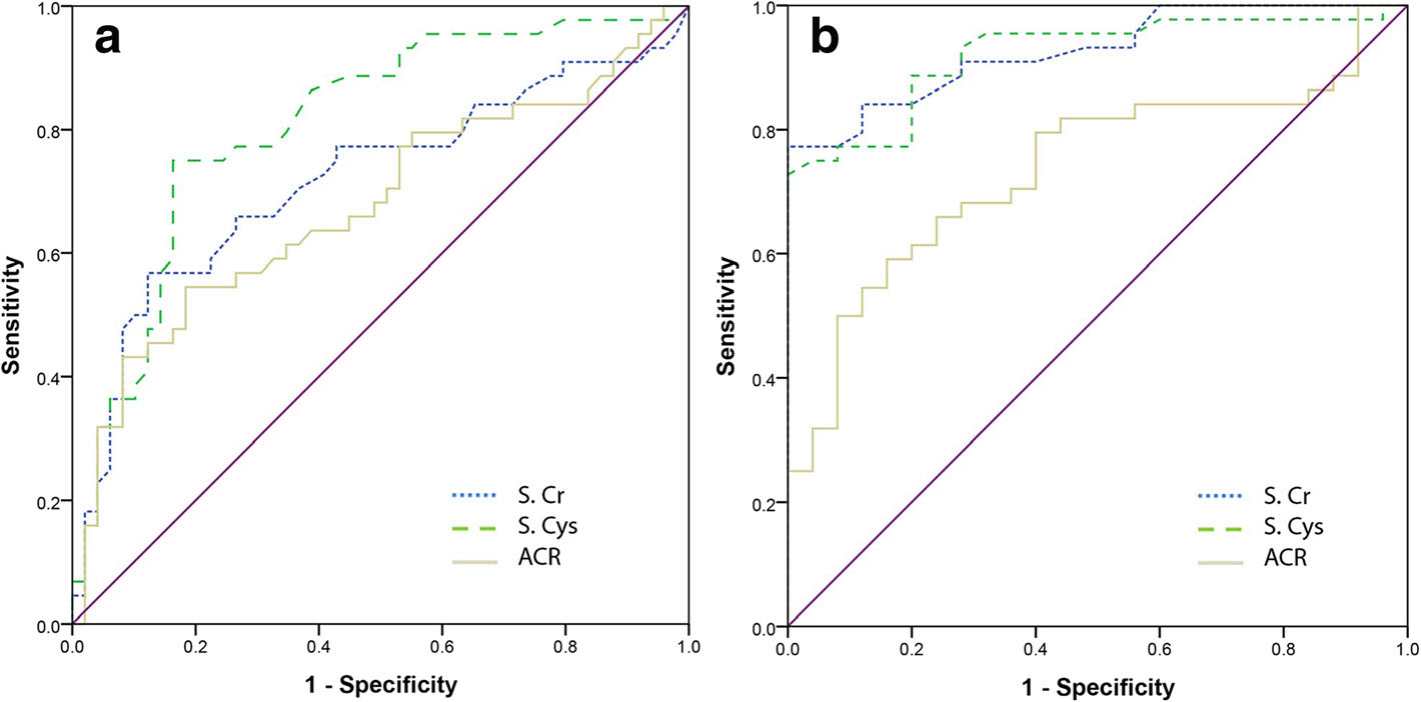
ROC plots for target markers: **a** CKDu against endemic control, **b** CKDu against non-endemic control

Figure 3 depicts simultaneous ROC plots generated for the target markers as: CKDu cases against endemic and non-endemic controls respectively. Table 4 denotes respective AUCs estimated for each plot with 95% CI.

**Table 4:** AUCs for target markers used in the differentiation of CKDu against endemic control and non-endemic control

	CKDu against non-endemic control (lower & upper bound at 95% CI)	CKDu against endemic control (lower & upper bound at 95% CI)
S.Cr	0.926 (0.868–0.984)	0.718 (0.610–0.827)
S.Cys	0.920 (0.857–0.984)	0.808 (0.718–0.898)
ACR	0.737 (0.619–0.855)	0.678 (0.566–0.790)

With respect to ROC plots of CKDu patients against endemic controls, highest AUC was observed for S.Cys followed by S.Cr and ACR. The percentage difference in AUCs of S.Cr and S.Cys was 11.3%. However, in ROC plots constructed against non-endemic controls, S.Cr exhibited the highest AUC followed closely by S.Cys. Both S.Cr and S.Cys seemed to perform similarly well in terms of AUC and the percentage difference in AUCs between S.Cr and S.Cys was only 0.6%. When AUCs were determined for a combined parameter consisting of S.Cr and ACR, slightly improved values than values of independent S.Cr were observed (CKDu vs non-endemic control: 0.927, CKDu vs endemic control: 0.725). Table 5 presents ﻿o﻿ptimal cutoff values ﻿for each examined marker with the best combination of corresponding Sn and Sp.

**Table 5:** Specificity (Sp), Sensitivity (Sn) and cutoff values of target biomarkers for CKDu cases against non-endemic and endemic controls

	CKDu against non-endemic control			CKDu against endemic control		
	Cutoff^a^	Sn	Sp	Cutoff^a^	Sn	Sp
S.Cr	89	0.84	0.88	111.5	0.57	0.88
S.Cys	1.01	0.89	0.80	1.22	0.75	0.84
ACR	6.06	0.70	0.64	12.66	0.54	0.82
Dipstick proteinuria^b^					0.4	0.8

^a^Cutoff values in same units as in Table 3

^b^dipstick proteinuria (≥1+ and including trace)

**Fig. 4:**
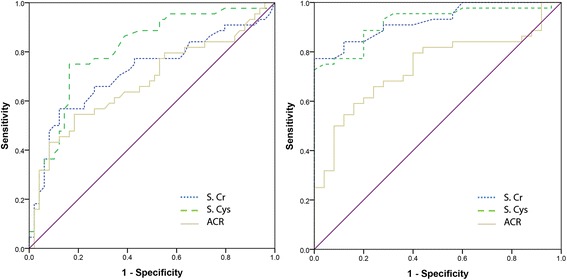
ROC plots for target markers: CKDu against CKD

When CKDu cases were analysed against non-endemic controls, Sn vales for S.Cr, S.Cys and ACR were higher by 32%, 16% and 23% respectively compared to when CKDu cases were analysed against endemic controls. Sp values of S.Cys and S.Cr were comparable in ROCs generated against both endemic and non-endemic controls. Interestingly for ACR, Sp was 22% lower against non-endemic controls. As ROCs could not be generated for non-continues results of dipstick proteinuria, manually calculated Sn and Sp values are denoted for comparison (Table 5). A ROC plot was generated to compare stage-matched (stage 2 – 4) CKDu and CKD patients which is depicted in Fig. 4.

The three target markers were evaluated on their ability to differentiate between CKDu and CKD. S.Cr and S.Cys both did not exhibit demarcation capabilities higher than the probability of pure chance. Interestingly, ACR exhibited an outstanding demarcation capability corresponding to a Sn of 0.74 and Sp of 0.83.

## Discussion

The global prevalence of diabetic and hypertensive CKD is considered to be around 8–16%. CKDu which has been observed in rural Sri Lanka, Central America and the Balkan region is becoming a global epidemic. For proper management of this disease, early detection and intervention is crucial.

Male predominance in CKDu has been identified by previous studies [12, 37, 38]. Due to the limited number of biopsy proven female patients among participants, biopsy proven male participants were prioritized. Healthy subjects were selected after exclusion of CKD/CKDu by means of clinical history, an examination and renal functional tests. CKDu exclusion by renal biopsy examination was not practiced on healthy individuals due to its invasive nature. CKDu patients of thisstudy had been diagnosed between 2009 and 2012 and that time interval between first diagnosis and recruitment was more than a year for each patient.

Dipstick proteinuria ≥1+ indicates that protein excretion is in the macro-albuminuria range (>300 mg/g-Cr). Biopsy-proven CKDu patients who showed micro or macro proteinuria or hypertension after diagnosis were treated with RAAS blockers and subsequently, proteinuria could have diminished. Rest of the CKDu cases, considered minimally-proteinuric, were not on RAAS blockers. According to available data, about 44% of the current biopsy proven CKDu cohort, of which this study group is a subset, is under RAAS medication. Majority of early stage CKDu cases were manifesting in the microalbuminuria range which could not be detected by dipstick proteinuria. This observation is corroborated by a study done in similar areas of Sri Lanka by Selvarajah et al. (2016), which support that early CKDu cases were mainly present with microalbuminuria range of proteinuria (30–300 mg/g-Cr). In an anti-proteinuric drug trial for CKDu patients, early CKDu cases (*n* = 130) were subjected to a course of Enalapril for 12 months and, as an outcome, the baseline ACR had reduced from 162 to 55.4 mg/g-Cr while the placebo group (*n* = 133) exhibited an increased ACR from 197.9 to 253.2 mg/g-Cr [39]. Since the albumin sensitivity of the dipstick test is >300 mg/g-Cr, the above changes due to the RAAS blocker could not have been detected by the dipstick test [40]. This corroborates on the reasons why an alternative kidney damage marker is required in place of dipstick proteinuria test for CKDu screening in Sri Lanka. Further, the micro-albumin range or below levels of ACR, manifestation in CKDu cases (Table 3) implied that albumin excretion by itself may not be a successful candidate marker for CKDu screening in Sri Lanka under current cutoff values. This is in agreement with the experience of clinicians in CKDu endemic regions of the country.

A notable observation was that higher AUCs were obtained for all three markers when CKDu patients were analysed against non-endemic controls. When compared to endemic controls, AUCs were distinctly lower (Table 4). This contrast in AUCs may reflect on the fraction of undetected patients through screenings in endemic areas by having negative dipstick proteinuria. In ROC plots for CKDu cases against endemic and non-endemic controls, a difference in cutoff limits exceeding 50% was observed for ACR meanwhile 20% and 17% for S.Cr and S.Cys respectively. Currently accepted ACR cut off is 30 mg/g-Cr as per standard guidelines. Cutoffs for ACR against endemic controls and non-endemic controls in CKDu fall substantially below 30 mg/g-Cr. This observation suggests that use of cutoff values derived from general population may not be accurate in CKDu endemic areas. A similar pattern was observed between S.Cr and S.Cys for which a positive Pearson’s correlation coefficient of 0.922 was observed. This corroborates Rule et al. (2006) who suggested the complementary behavior of S.Cys to S.Cr [29]. In the attempt of distinguishing non-endemic controls, the effectivity of functional markers: S.Cr and S.Cys show similar results as a screening tool for CKDu. However, when demarcating against endemic controls S.Cys was observed to be superior. When CKDu cases were analysed against CKD cases by means of a ROC plot (Fig. 4), only ACR expressed a separation capability with an acceptable Sp and Sn. This observation clearly suggests that ACR may be able to detect the differences in albumin excretion patterns between CKD and CKDu patients

In clinical practice, kidney damage markers such as ACR have predominantly been used complementary with serum markers such as creatinine and cystatin C. A direct comparison between these two types was beyond the scope of this study. Rather, an evaluation of the ROC-based case-control demarcation capability of each type was targeted. As CKDu features interstitial damage represented by tubular atrophy, filtered albumin which is unable to be reabsorbed by tubules should appear in the urine [38]. Theoretically, the albumin excretion should be proportionate to the degree of renal damage. However, in the actual situation, ACR exhibited a sub-par sensitivity for CKDu patients against both control groups and S.Cr and S.Cys emerged to be superior. This observation is unique concerning CKDu in Sri Lanka and complies with the current knowledge of CKDu as a minimally-proteinuric disease in comparison to CKD. It further suggests that screening with dipstick proteinuria may result in poor detection.

The restricted number of CKDu cases and controls is a limitation of this study. The total number of biopsy proven CKDu cases (male and female) identified for the study were less than 100 due to lack of patients' consent for the biopsy test. Among 51 patients who participated, 44 were male forcing the exclusion of female cases which led to a noticeable depletion in the original cohort size of patients and controls in the study. Limited biopsied cases further affected this study by enforcing the recruitment of already intervened and non-intervened cases together. Already intervened cases under RAAS blockers may have interfered with the absolute discriminating ability of the target biomarkers. Intervened, biopsied cases were justified over un-intervened cases in this study. This was mainly due to the unavailability of internationally accepted case demarcation parameters defined for CKDu. Despite the fact that novel tubular proteins have commendable capabilities, they were not employed for this study. Respective reasons were limited experience, technical difficulties, high cost and infeasibility as field tests. Distinguishing total protein rather than albumin through a protein detection test (protein: creatinine ratio) may have been a competitive alternative, but not utilized due to lower sensitivity and high false positive rates.

## Conclusions

CKDu in rural Sri Lanka has started to reach epidemic proportions. High cost associated with management of end stage renal failure due to CKDu has substantially impacted both rural and the national economy. The credibility of screening CKDu by means of dipstick proteinuria, which is the current method, has been questioned due to its limited sensitivity, subjectiveness and high probability for human error.

No previous studies have assessed the sensitivity and specificity of currently used CKD screening markers for CKDu in Sri Lanka. This study emphasizes the limitations of using dipstick proteinuria for screening CKDu in Sri Lanka while investigating strengths and weaknesses of S.Cys, S.Cr and ACR. It is obvious that S.Cys is the best functional marker to distinguish CKDu cases from healthy subjects in mass screening programs. The high cost of S.Cys could be unfavorable in practice. Therefore, as an appreciably accurate and a cost-efficient functional marker: S.Cr along with ACR: a renal damage marker, could be used for successful detection of CKDu cases in mass screenings. Due to the inferior sensitivity against endemic population, ACR does not seem to be favorable as an individual substitute marker. Moreover, when identifying CKDu patients in disease-endemic regions, contrasting ROC-based cutoff levels against endemic and non-endemic controls suggested that using cutoff values derived from general population may not be accurate for an endemic population. Given the minimally proteinuric nature of CKDu, lowering the current ACR cutoff limit below 30 mg/g-Cr may be a viable option to improve detection of CKDu cases. However, extensive clinical investigations are needed before such measures are implemented. Further studies, involving larger study samples and more biomarkers, are greatly needed to conclusively elucidate and fine tune an optimal screening tool for accurate identification of CKDu patients in Sri Lanka.

## Abbreviations

ACR: Albumin to creatinine ratio
AUC: Area under curve
CI: Confidence Interval
CKDu: Chronic kidney disease of uncertain aetiology
eGFR: estimated Glomerular Filtration Rate
ESRD: End Stage Renal Disease
MDRD: Modification of Diet for Renal Disease
NCP: North Central Province
RAAS: Renin Angiotensin- Aldosterone System
ROC: Receiver operating characteristic
S.Cr: Serum creatinine
S.Cys: Serum cystatin C
SD: Standard deviation
Sn: Sensitivity
Sp: Specificity
