# Erratum to: screening for chronic kidney disease of uncertain aetiology in Sri Lanka: usability of surrogate biomarkers over dipstick proteinuria

**DOI:** 10.1186/s12882-017-0642-2

**Published:** 2017-07-14

**Authors:** BMC Nephrology

**Affiliations:** London, UK

## Erratum

In the original publication of this article [1], figure 4 is incorrect. In this Erratum the original figure 4 (Fig. 1) and the correct figure 4 (Fig. 2) are published. The original article has been updated.

**Fig. 1:**
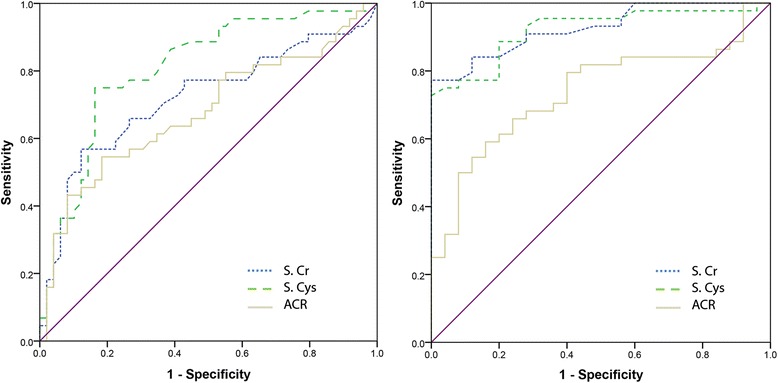
Original version of figure 4 as published on 19 June 2017

**Fig. 2:**
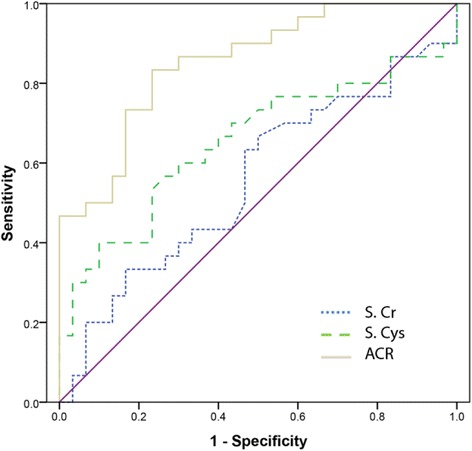
Corrected version of figure 4

The publisher apologizes for the inconvenience caused to the authors and readers.
